# The Effect of Anxiolytics on Tinnitus

**DOI:** 10.3390/jcm12227076

**Published:** 2023-11-14

**Authors:** George Psillas, Chrysoula Vlachou

**Affiliations:** 1 1st Otolaryngology Department, Faculty of Health Sciences, School of Medicine, Aristotle University of Thessaloniki, AHEPA Hospital, 1, Stilponos Kyriakidi St., 546 36 Thessaloniki, Greece; 2 Faculty of Health Sciences, School of Medicine, Aristotle University of Thessaloniki, AHEPA Hospital, 1, Stilponos Kyriakidi St., 546 36 Thessaloniki, Greece

Tinnitus is a perceptual disorder in which sound is perceived by the patient in the absence of an external or internal acoustic stimulation. Subjective or idiopathic tinnitus is observed in almost 10–14% of the general population and its prevalence rises as patients get older, reaching its highest point at approximately 60–70 years of age [1]. Various methods are currently used to treat tinnitus, including medication, cognitive behavioral therapy, tinnitus retraining therapy, transcranial magnetic stimulation, neuromodulation, sound therapy and hearing aids.

Psychiatric symptoms, such as anxiety and depression, are very frequent among patients who experience tinnitus [2]. Inversely, tinnitus symptoms have been linked to a variety of psychological and psychosomatic symptomatology, with a high prevalence of comorbid depressive disorders among individuals seeking assistance for tinnitus [3]. Although the auditory cortex is not implicated in anxiety disorders, both neural pathways between tinnitus and anxiety disorders include networks as a part of the limbic system, with the amygdala representing a highly significant structure in this context.

Anxiolytics, such as benzodiazepins, are among the most prescibed medicines for tinnitus, while no medication has been approved as a treatment for tinnitus by the US Food and Drug Administration [4] (Table 1). It has been hypothesised that tinnitus perception may arise, in part, from a spontaneous neural activity increase in the central auditory system [5]. Benzodiazepines potentiate the inhibition caused by the release of γ-aminobutyric acid (GABA). Thus, if tinnitus is due to auditory central nervous system hyperactivity, then it is likely that benzodiazepines reduce tinnitus intensity by decreasing this excessive activity through enhancing GABA-mediated inhibition [6].

However, a systematic review revealed diverse outcomes in the effects of different benzodiazepins on tinnitus. After clinical trials were conducted, the final conclusion underlined that there was no robust evidence supporting the use of benzodiazepins [7]. In addition, according to the guidelines of the American Academy of Otolaryngology—Head and Neck Surgery [8], anxiolytics were not recommended for the management of tinnitus, as clinical trials did not present a positive effect. It has also been demonstrated that high doses of benzodiazepines can prevent neuroplasticity, reducing the ability of the brain’s natural process of adapting to the constant sound of tinnitus [9]. Moreover, as tinnitus is a long-term condition, the use of short-term medication, such as benzodiazepines, is unlikely to be the best option, and the risk is higher. Care must be taken when the usage of benzodiazepines is discontinued as withdrawal can even trigger new tinnitus [10]; in the case study reported in [10], severe tinnitus interfered with sleep and concentration and worsened anxiety.

The question is whether patients suffering from co-morbid psychiatric symptoms (e.g., anxiety, depression, insomnia) may benefit from anxiolytic medications for their tinnitus. Due to the co-occurrence of anxiety and tinnitus, it is assumed that taking anti-anxiety medications would be a good option for managing both conditions. This could be the case in short-term dosage, due to the general anxiolytic effect of benzodiazepines rather than the direct effect on the neurophysiological cause of tinnitus [7,11]. In chronic tinnitus (lasting at least 3 months), the single administration of anxiolytics is not suggested, as they may worsen the intensity of tinnitus and prolong the duration of the symptoms.

Tinnitus is also linked with sleep disorders, including insomnia, causing difficulties in both the start-up and maintenance of sleep. This state may lead to an overall reduction in quality of sleep [12]. Due to their anxiolytic and sleep-inducing properties, benzodiazepines should be expected to have calming effects on comorbid anxiety and insomnia. Nevertheless, anxiolytics are questioned in patients with chronic or severe tinnitus [13], and therapeutical options such as cognitive behavioral therapy [12] are alternative recommendations.

Based on the above observations, anxiolytics could be beneficial for tinnitus in terms of short-term effects; however, they should be contrandicated for chronic or severe tinnitus, mainly in patients with co-morbid psychiatric symptoms. Benzodiazepines have been reported to have a significant side-effect profile, with a high risk for abuse and dependence [7].

Further clinical studies evaluating the neurobiology of and pharmacology for tinnitus are warranted, and in this direction, our Special Issue encourages submissions to address these topics. Multicentric studies and collaboration are required to establish new therapeutical protocols and treatment options. Assessing the current knowledge and elucidating the neuromodulation pathways and pathophysiologic mechanisms for tinnitus will help us to promote our undestanding of this complex clinical symptom.

## Figures and Tables

**Table 1:** Off-label anxiolytic drugs in the treatment of tinnitus.

Alprazolam	Diazepam
Biomazepam	Amylobarbitone
Flunitrazepam	Zolpidem
Oxazepam	Protriptyline
Lorazepam	Clonazepam
